# Supplementary material for: The interactome of intact mitochondria by cross-linking mass spectrometry provides evidence for coexisting respiratory supercomplexes
Source: Mol Cell Proteomics. 2017 Dec 8;17(2):216–32. doi: 10.1074/mcp.RA117.000470 (PMC5795388; doi:10.1074/mcp.RA117.000470)
Supplement: Supplemental Data [file supp_RA117.000470_133922_0_supp_23978_fzffwf.zip › spectra_annotation/mito_spectra_annotation/39-1-3-1-2-1.pdf]

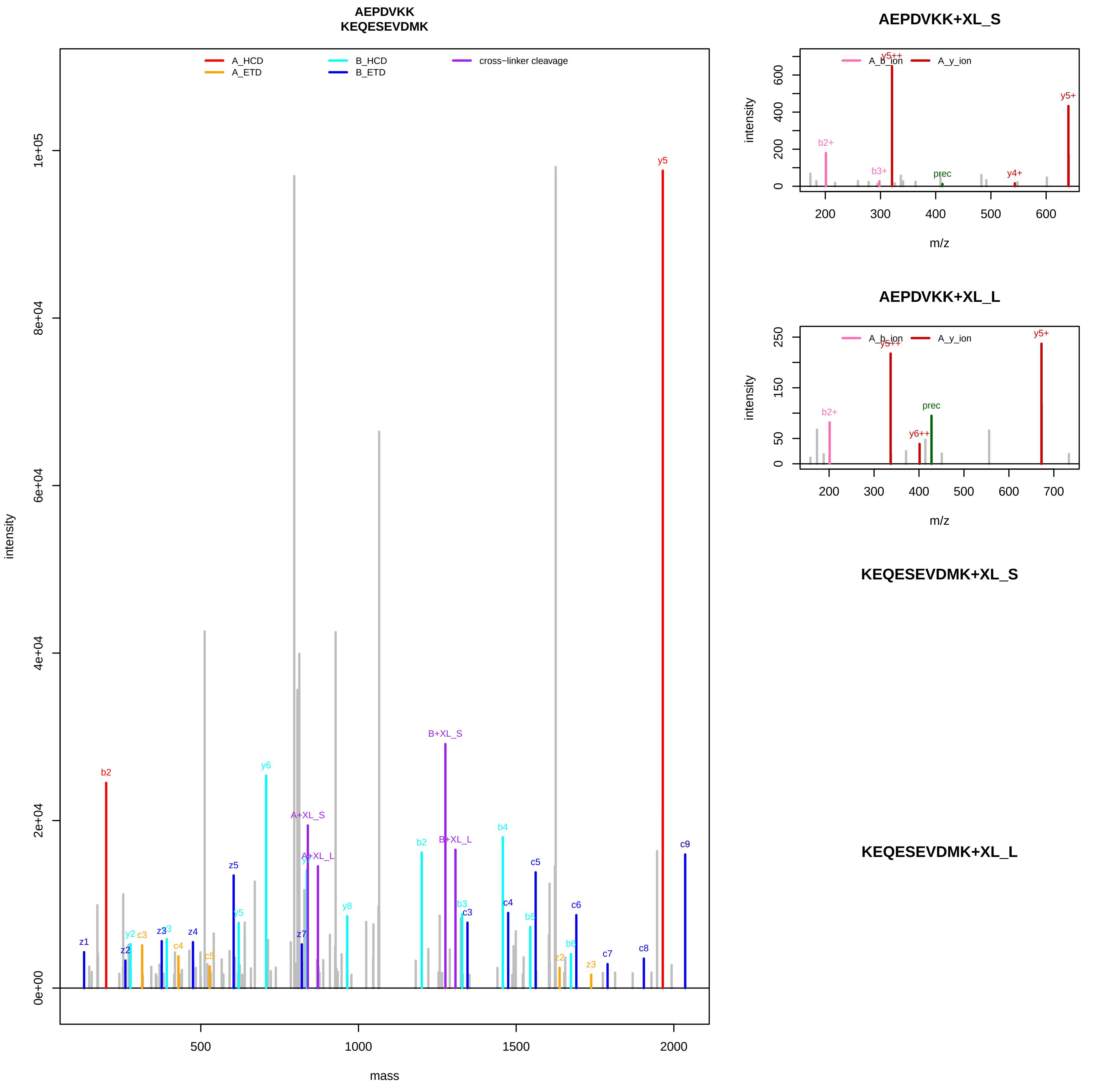
AEPDVKK
KEQESEVDMK
A_HCD
A_ETD
B_HCD
B_ETD
cross−linker cleavage
intensity
mass
y5
b2
y6
B+XL_S
A+XL_S
A+XL_L
B+XL_L
b2
b4
c9
c5
z5
AEPDVKK+XL_S
A_b_ion
A_y_ion
y5++
y5+
b2+
b3+
prec
y4+
intensity
m/z
AEPDVKK+XL_L
A_b_ion
A_y_ion
y5++
y5+
b2+
y6++
prec
intensity
m/z
KEQESEVDMK+XL_S
KEQESEVDMK+XL_L
